# B Cell Recognition of *Candida albicans* Hyphae *via* TLR 2 Promotes IgG1 and IL-6 Secretion for T_H_17 Differentiation

**DOI:** 10.3389/fimmu.2021.698849

**Published:** 2021-11-08

**Authors:** Marta Ferreira-Gomes, Melissa Wich, Sally Böde, Bernhard Hube, Ilse D. Jacobsen, Berit Jungnickel

**Affiliations:** ^1^ Institute of Biochemistry and Biophysics, Faculty of Biological Sciences, Friedrich Schiller University, Jena, Germany; ^2^ Department Microbial Pathogenicity Mechanisms, Leibniz Institute for Natural Product Research and Infection Biology, Hans Knöll Institute, Jena, Germany; ^3^ Institute of Microbiology, Faculty of Biological Sciences, Friedrich Schiller University, Jena, Germany; ^4^ Research Group Microbial Immunology, Leibniz Institute for Natural Product Research and Infection Biology, Hans Knöll Institute, Jena, Germany

**Keywords:** *Candida albicans*, B cells, toll-like receptors, IL-6, TH17 cells, fungal cell wall

## Abstract

*Candida albicans* is usually a benign member of the human gut microbiota, but can become pathogenic under certain circumstances, for example in an immunocompromised host. The innate immune system, in particular neutrophils and macrophages, constitutes a crucial first line of defense against fungal invasion, however adaptive immunity may provide long term protection and thus allow vaccination of at risk patients. While T_H_1 and T_H_17 cells are important for antifungal responses, the role of B cells and antibodies in protection from *C. albicans* infection is less well defined. In this study, we show that *C. albicans* hyphae but not yeast, as well as fungal cell wall components, directly activate B cells *via* MyD88 signaling triggered by Toll- like receptor 2, leading to increased IgG1 production. While Dectin-1 signals and specific recognition by the B cell receptor are dispensable for B cell activation in this system, TLR2/MyD88 signals cooperate with CD40 signals in promoting B cell activation. Importantly, recognition of *C. albicans via* MyD88 signaling is also essential for induction of IL-6 secretion by B cells, which promotes T_H_17 polarization in T-B cell coculture experiments. B cells may thus be activated directly by *C. albicans* in its invasive form, leading to production of antibodies and T cell help for fungal clearance.

**Graphical Abstract:**
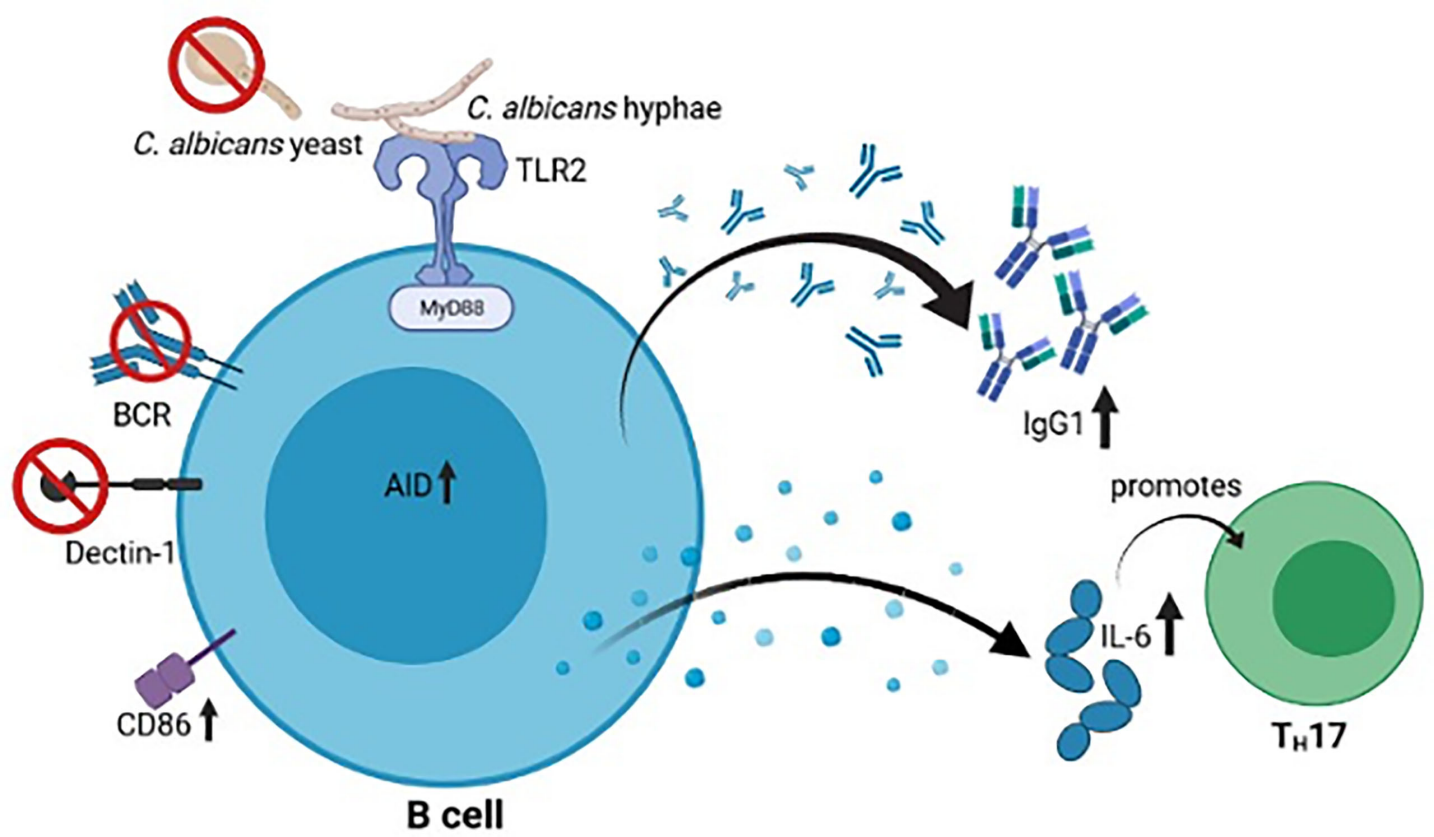
Upon MyD88- and TLR-mediated recognition of *C. albicans* hyphae, but not yeasts, murine B cells show an increased activation (CD86 and AID) and secretion of IgG1 as well as IL-6, which promotes TH17 differentiation. The figure was created with Biorender.com.

## Introduction

*Candida albicans* is a commensal fungus that colonizes mucosal tissues, being part of the normal microbiota of most healthy individuals. However, this benign commensal can also cause severe infections in immunocompromised hosts, being a common cause of nosocomial infections (1, 2). Despite increasing research efforts in recent years, compared to other infections, fungal infections are still understudied and often misdiagnosed, leading to a high mortality rate (3).

One characteristic feature of *C. albicans* that plays a crucial role during its commensal or pathogenic life style is the fact that it can grow in different morphological forms: an ellipsoid shaped yeast form or an elongated filamentous hyphal form (4). Both morphologies are found during infection and expose different and characteristic cell wall components and proteins on their surfaces. However, the hyphal form has been shown to be the invasive morphology, being essential for penetration through host barriers (5, 6). This difference in morphology also leads to differential recognition by host cells and has been shown to be critical for host discrimination between commensal and pathogenic cells (7–12).

The cell wall of *C. albicans* can be differentiated into two layers, the outer layer, mainly composed of N- and O-linked mannans, and the inner layer, composed of β-glucans and chitin. Cell wall proteins in both layers are mostly bound to chains of β-1,6-glucans (13, 14). These different components contain pathogen-associated molecular patterns (PAMPs) that are recognized by pattern recognition receptors (PRRs) on the host cells. PRRs are differentially expressed in different cell types, accounting for the specialized function displayed upon encountering specific pathogens. Several PRRs are involved in recognizing *C. albicans*, including Toll-like receptors (TLRs) and C-type lectin receptors (CLRs) (15). The C-type lectin receptor Dectin-1 recognizes β-1,3-glucans, leading to, among others, the activation of the canonical and non-canonical NF-κB pathways *via* Syk (16, 17). Toll-like receptor-mediated recognition of PAMPS also leads to NF-κB activation and the activation of other signaling pathways (18). TLR2 and TLR4 recognize phospholipomannan and O-mannan on the fungal surface, respectively (19, 20), and signal *via* the interaction of the cytoplasmic TIR domains with the adapter protein myeloid differentiation primary response gene 88 (MyD88). Subsequent NF-κB activation *via* TRAF-6 triggers multiple effects in immune cells, among them increased cytokine production, capable of influencing T cell differentiation (21, 22). Dectin-1 and TLR2 can also recognize Zymosan, a cell wall preparation from *Saccharomyces cerevisiae* widely used as a model for fungal immune stimulation (20).

The role of innate immunity in defense against *C. albicans* has been widely studied, with a focus on neutrophils and macrophages as the most important effector cells (15, 23, 24). Regarding adaptive immunity, T helper 17 (T_H_17), and to a minor extent, T helper 1 (T_H_1) cells are the principal cell types mounting an effective response towards *C. albicans* (25–27). Previous studies on patients with immunoglobulin deficiencies or on B-cell deficient mice suggested that B cells play no major role in *C. albicans* infection. However, more recent studies may change this view, now proposing that B cells and antibodies are in fact involved in multiple protective mechanisms during *C. albicans* infection (28–36).

B cell activation occurs both dependently and independently of T cell help. In a T cell-dependent activation, the B cell recognizes a specific antigen through its B cell receptor (BCR) while T helper cells provide additional stimulation by CD40 ligand-CD40 interaction. T cell independent activation can further be divided into two types: type 1, where B cells are activated by recognizing PRRs *via* their TLRs and type 2, where activation occurs by extensive crosslinking between BCRs (mostly for polysaccharides). Recent studies suggest that innate cells can also be involved in both T-dependent and -independent B cell activation (37). Noteworthy is the fact that TLR signaling synergizes with both BCR and CD40 signaling to enhance B cell activation (38, 39). Upon activation, and depending on localization, B cells can undergo somatic hypermutation for production of high affinity antibodies and/or class switch recombination for production of antibodies with different effector functions. Both processes depend on the activity of activation-induced cytidine deaminase (AID) (40). Another consequence of B cell activation is the production of cytokines. Depending on the provided stimulation, B cells are known to produce pro-inflammatory cytokines (IL-6, TNF-α) or the anti-inflammatory cytokine IL-10, therefore influencing other cell types (41–43).

This study shows that *C. albicans* hyphae, but not yeast cells, and fungal cell wall components (Zymosan) can activate B cells mostly *via* TLR recognition. This activation is in turn translated into an increased production of antibodies and an increased secretion of the cytokine IL-6, which supports T_H_17 polarization, providing a functional link between B cell responses to *C. albicans* and protective T cell responses.

## Material and Methods

### Preparation of Heat-Killed *Candida albicans*

*Candida albicans* yeast and hyphae from strain SC5314, *C. glabrata* yeast from strain ATCC2001 and *C. auris* yeast from the strain B8441 were grown and heat-killed for cell culture stimulation. Briefly, *C. albicans, C. glabrata and C. auris* from a YPD (1% yeast extract, 2% glucose, 2% peptone) culture were inoculated in YNB (0,67% yeast nitrogen base, 1% glucose) at 30°C, 180rpm for yeast growth and in YNBNP (0,67% yeast nitrogen base, 0.2% glucose, 5mM N-acetylglucosamine in 25mM potassium phosphate buffer pH7) at 37°C, 180rpm for hyphal growth for 24 hours. Cultures were then washed and set to a density of 10^8^ yeast cells/mL in PBS. The same dilution factor was used for hyphal cells. Diluted cells were distributed into 1.5mL tubes and incubated for 15min at 80°C, 800rpm. After ensuring cell death by plating of aliquots on YPD agar plates and incubation for 48 to 72 hours at 37°C, cells were centrifuged, weighed and their concentration set to 100mg/mL in PBS. Cells were stored at -20°C. To reduce cell clumping, hyphae cells were sonicated prior to usage.

### Mouse B Cell Isolation and Stimulation

All mouse experiments were approved by the appropriate institutional and governmental review committees for animal welfare (Thüringer Landesamt für Verbraucherschutz; breeding license 02-052/16). All mouse lines were on a C57BL7/6 background. Primary B cells were isolated from spleens of 8- to 16-week-old mice. Briefly, single cell suspensions were prepared from spleens, depleted of red blood cells using Red Blood Cell Lysis Buffer (Sigma) and purified by negative selection using MACS with anti-CD43 beads (Miltenyi Biotec) according to the manufacturer’s instructions. Isolated B cells were then cultured at 5x10^5^ cells/mL in 24-well plates for 5 days at 37°C in RPMI (Invitrogen) supplemented with 10mM HEPES (ThermoFisher), 50mM β-mercaptoethanol (Sigma), 100U/mL Pen Strep (Gibco) and 10% FBS (Sigma) (cRPMI). Stimulating conditions were achieved by adding 10µg/mL LPS (Sigma, L4391) or 1µg/mL anti-CD40 (eBioscience) with 20ng/mL IL-4 (eBioscience). 3 to 4 hours after seeding, 30µg/mL Zymosan (Wako) or 500µg/mL heat-killed *Candida albicans* yeast or hyphae were added to the culture. Cultured cells were fed at day 3, maintaining the same culture conditions. After 3 and 5 days of culture, cells were harvested and supernatants stored at -20°C and -80°C for further analysis.

### Human B Cell Isolation and Stimulation

All human experiments were conducted in accordance with the Declaration of Helsinki and were approved by the ethics committee of the University hospital Jena. Human B cells were isolated from buffy coats of healthy donors. Briefly, PBMCs were extracted from the blood by gradient centrifugation using Biocoll-Plaque (density 1.077 g/mL). PBMCs were depleted of thrombocytes by two washing steps (centrifugation with 200g for 10 min at 4°C). B cells were purified by negative selection using MACS with the human B cell isolation kit II (Miltenyi Biotec) according to the manufacturer’s instructions. Isolated B cells were cultured at 2x10^6^ cells/mL in 96-well U-bottom plates for 6 days at 37°C in RPMI (Invitrogen) supplemented with 2 mM glutamine (Gibco), 1 mM pyruvate (Gibco), 100 µg/µL Pen Strep (Gibco) and 10% FBS (Sigma). For stimulation, 5 µg/mL anti-IgA/IgG/IgM (Jackson ImmunoResearch), 0.5 µg/mL (low anti-CD40 conditions) or 1 µg/mL (high anti–CD40 conditions) anti–;CD40 (R&D Systems), 50 ng/mL IL-2 (ImmunoTools) and 50 ng/mL IL-10 (ImmunoTools) were added. 3 hours after seeding, 30µg/mL Zymosan (Wako) or 500µg/mL heat-killed *Candida albicans* yeast or hyphae were added to the culture to achieve a final volume of 100 µL. After 3 days of culture, supernatants were recovered and stored at -20°C for further analysis. Cells were resuspended as at day 0, but without anti-CD40 and anti-IgA/IgG/IgM stimulation. After an additional 3 days of culture, supernatants were recovered and stored at -20°C for further analysis.

### B Cell Flow Cytometry Analysis

In all experiments, flow cytometry was performed using a LSR Fortessa cytometer (BD Biosciences) and the results were analyzed using FlowJo software (FlowJo, LLC). To assess B cell purity upon isolation, isolated cells were stained with anti-CD3 and anti-B220 (mouse, BD Biosciences) or anti-CD19 (human, BioLegend) and analyzed by flow cytometry. To assess class switching to IgG1 on mouse B cells, after 3 and 5 of culture cells were stained with anti-B220 and anti-IgG1 (BD Biosciences) and analyzed by flow cytometry. In experiments involving AIDCre-Rosa26YFP mice, cells were stained with anti-B220 (BioLegend) and anti-IgG1 (BD Biosciences), and the percentage of YFP-positive cells was analysed in addition. To analyze the expression of CD86 on mouse B cells, B cells were cultured for 6 and 8 hours and subsequently stained with anti-B220 (BD Biosciences) and anti-CD86 (eBioscience) or its IgG2b κ isotype control (ThermoFisher). To investigate Dectin-1 expression in B cells, splenic cells were stained with anti-B220 (BD Biosciences) and anti-Dectin-1 (clone 2A11, Thermo Fisher Scientific and clone bg1fpj, eBioscience). In all cases DAPI (Sigma) was added to the cells prior to analysis for dead cell exclusion. To assess the intracellular IL-6 in B cells, after 5 days of culture cells were treated with 10 ng/mL PMA (Sigma) and 1 µg/mL ionomycin (Sigma), followed by incubation in a PMA/ionomycin solution containing 50 µg/mL Brefeldin A (BioLegend). After fixation with 2% formaldehyde (Roth), cells were permeabilized using a 5% Saponin (Sigma) solution to enable intracellular cytokine staining. Cells were labelled with anti-CD3, anti-B220 (BD Bioscience) and anti-IL6 (BioLegend) and analyzed by flow cytometry.

### ELISA

B cell culture supernatants were analyzed by ELISA to determine the concentration of secreted IgM, IgG1 (mouse) or IgG (human). Briefly, 96-well plates (Nunc, Maxisorp) were coated with capture antibody overnight at 4°C. After washing the plate and blocking unspecific binding, supernatants were incubated for 1 hour at room temperature and were detected using a biotin-coupled antibody, streptavidin-HRP and O-phenylenediamine substrate (Sigma). After stopping the reaction with 3N HCL, absorbance at 492nm was measured. Immunoglobulin concentration was determined by comparison with a standard curve. For mouse IgM and IgG1 detection the antibodies anti-IgM, anti-IgG1, anti-IgM-biotin, anti-IgG1-biotin, purified IgM and purified IgG1 were all purchased from BD Pharmingen. For human IgG detection the antibodies anti-IgG and anti-IgG-biotin were purchased from BD Pharmingen and purified IgG was purchased from Jackson ImmunoResearch.

IL-6 and IL-10 concentration in mouse B cell culture supernatants was measured using the kits Mouse IL-10 ELISA Ready-SET-Go! and Mouse IL-6 ELISA Ready-SET-Go! (eBioscience). ELISA was performed according to the manufacturer’s instructions.

### Western Blot

B cells isolated from murine spleens were cultured at 5x10^5^ cells/mL in 6-well plates at 37°C in cRPMI. Stimulation conditions were achieved by adding 1µg/mL anti-CD40 (eBioscience) with 20ng/mL IL-4 (eBioscience) into the medium. After seeding, either 10µg/mL LPS (Sigma, L4391), 30µg/mL Zymosan (Wako) or 500µg/mL heat-killed *Candida albicans* yeast or hyphae were added. After 12 hours of incubation, the B cells were harvested. B cells lysates were prepared by incubation in 20 mM HEPES pH 7.9 (Invitrogen), 350 mM NaCl (Carl Roth), 20% glycerin (Carl Roth), 1 mM MgCl_2_ (Carl Roth), 0.5 mM EDTA (AppliChem), 0.1 mM EGTA (Fluka Chemie), 1% NP-40 (AppliChem) and protease and phosphatase inhibitors (Roche, Basel, Switzerland) for 20 min at 4°C. The protein concentration of the lysates was measured using the colorimetric DC Protein Assay (Bio-Rad). 30 µg of lysate proteins were diluted in sample buffer containing 20% glycerol (Carl Roth), 4% sodium dodecyl sulfate (Carl Roth), and 120 mN Tris pH 6.8 (Carl Roth) and 0.1% bromophenol blue (Carl Roth). Prior to the sodium dodecylsulfate polyacrylamide (12% acrylamide, Carl Roth) gel electrophoresis, 2 µL of 1 mM DTT (Invitrogen) was added and samples were denatured at 95°C for 5 min. Blotting onto a polyvinylidene fluoride membrane (Carl Roth) was achieved with a wet tank transfer system (Hoefer) and its efficiency was confirmed by staining the membrane with Ponceau S solution (Sigma-Aldrich). The blocking of unspecific binding sites on the membrane and the incubation with antibodies was performed in Tris-buffered saline [50 mM pH 7.5 Tris-Cl (Carl Roth), 150 mM NaCl (Carl Roth) and 0.2% Tween20 (Carl Roth)] with 5% milk powder (Carl Roth). For protein detection, the membranes were incubated with the primary antibodies α–vinculin (BIOZOL), α-IRAK4 (Cell Signaling) or α-pIRAK4 (Abnova) and the secondary horseradish-peroxidase-conjugated antibodies α-mouse IgG (Promega), α-rabbit IgG (Cell Signaling) or α-mouse IgG (Cell Signaling), respectively.

### B Cell Proliferation

To follow B cell proliferation, isolated mouse B cells were stained with Vybrant CFDA SE Cell Tracer Kit (Thermo Fisher Scientific) during culture in different stimulating conditions. Briefly, after isolation, cells were set to 5x10^6^ cells/mL in prewarmed PBS and stained with 1µM CFDA SE for 10min at 37°C. After staining, the stain was quenched by addition of 5 times cold cRPMI. Cells were then re-pelleted, resuspended in prewarmed cRPMI and incubated for an additional 20min at 37°C to ensure complete modification of the probe. Following incubation, cells were re-pelleted and set in the appropriate conditions for stimulation and culture. CFSE dilution was analyzed by flow cytometry on days 1 to 4 after culture. In addition, cells were stained with anti-B220 (BD Biosciences) and DAPI (Sigma) for dead cell exclusion. Number of divisions during the analyzed time period was calculated by $Log_{2}\left(\frac{MFI_{d1}}{MFI_{d4}}\right)$ . MFI, mean fluorescence intensity.

### RT-PCR

RNA from mouse splenic cells, isolated B cells and the murine macrophage cell line RAW 264.7 was isolated using the Quick-RNA Miniprep kit (Zymo Research) according to the manufacturer’s instructions. RNA purification was confirmed by electrophoresis, by running the isolated RNA in a 1% agarose gel. cDNA synthesis was performed using the First Strand cDNA Synthesis Kit for RT-PCR (Roche) according to the manufacturer’s instructions. To detect clec7A/dectin-1 mRNA, the following pair of intron-spanning primers was designed: 5’-ACCACAAGCCCACAGAATCAT-3’ and 5’- GACTTGAAACGAGTTGGGGAAG-3’, amplifying a product of 347 base pairs. The primer pair 5’-ACCTTCAACACCCCAGCCAATGTACG-3’ and 5’-CTAATCCACATCTGCTGGAAAGATGG-3’ was used to detect β-actin as a loading control. These primers generated a product of 698 base pairs.

### Cytokine Screening Using LEGENDplex™

B cell culture supernatants were analysed for the presence of the cytokines TNF-α, IFN-γ, IL-2, IL-5, IL-4, IL-6, IL-10 and IL-13 using the bead-based immunoassay LEGENDplex™ (BioLegend). The assay was conducted according to the manufacturer’s instructions. Flow cytometry was performed using a LSR Fortessa cytometer (BD Biosciences) and the results analysed using the LEGENDplex™ Data Analysis Software (BioLegend) and Microsoft Excel.

### B-T Co-Culture and Flow Cytometry Analysis for IL-17 Expression

Primary B and T cells were isolated from spleens of 8- to 16-week-old mice. Recovery of splenic cells and isolation of B cell was performed as described above. Naïve CD4^+^ T cells were purified from the splenic cell suspension by depletion of non-CD4^+^ cells and CD4^+^ memory T cells *via* negative selection using MACS (Miltenyi Biotec) according to the manufacturer’s instructions. Isolated B and T cells were then co-cultured at 5x10^5^ cells/mL in 48-well plates for 4 days at 37°C in RPMI (Invitrogen) supplemented with 10mM HEPES (ThermoFisher), 50mM β-mercaptoethanol (Sigma), 100U/mL Pen Strep (Gibco) and 10% FBS (Sigma) (cRPMI). Stimulating conditions were achieved by adding 1µg/mL anti-CD3e (Biolegend) and 10 µg/mL anti-IgM (Jackson ImmunoResearch). Samples treated with a neutralizing anti-IL-6 antibody were additionally supplemented with 10 µg/mL anti-IL-6 (BD Bioscience). After seeding, 30µg/mL Zymosan (Wako) or 500µg/mL heat-killed *Candida albicans* hyphae or a TH17 differentiation inducing cocktail (10 µg/mL anti-IL-4 (Biolegend), 10 µg/mL anti-INF-γ (Biolegend), 10 µg/mL anti-IL-12 (Biolegend), 2 ng/mL TGF-β1 (New England Biolabs), 10 ng/mL IL-6 (Biolegend)) were added to the culture. After 4 days of culture, cells were treated with 10 ng/mL PMA (Sigma) and 1 µg/mL ionomycin (Sigma), followed by incubation in a PMA/ionomycin solution containing 50 µg/mL Brefeldin A (Biolegend). After fixation with 2% formaldehyde (Roth), cells were permeabilized using a 5% Saponin (Sigma) solution to enable intracellular staining.

In all experiments, flow cytometry was performed using a LSR Fortessa cytometer (BD Biosciences) and the results were analyzed using FlowJo software (FlowJo, LLC). To assess B and T cell purity upon isolation, isolated cells were stained with anti-CD3, anti-CD4 and anti-B220 (mouse, BD Biosciences) and analyzed by flow cytometry. Purity was <95% in all experiments. To analyze the expression of IL-17, after 4 of culture cells were stained with anti-B220, anti-CD4 and anti-IL-17-A (Biolegend) and analyzed by flow cytometry. An IgG1 κ isotype control antibody (BD Bioscience) was used to confirm the IL-17 staining.

### Statistical Analysis

Statistical significance was determined using two-tailed paired or unpaired t tests. Statistical analysis was performed using Sigma Plot 14.0 and Microsoft Excel Software. p values under 0.05 were considered significant (*p<0.05, **p<0.01, ***p<0.001).

## Results

### Increased Activation of Murine B Cells Upon Stimulation With *Candida albicans* Hyphae

**Figure 1:**
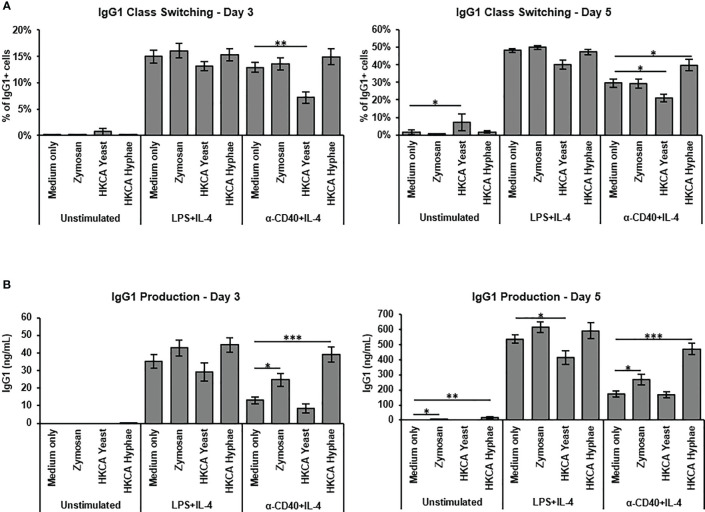
B cell stimulation with Zymosan and HKCA hyphae leads to increased antibody production. Splenic mouse B cells were cultured either unstimulated or stimulated with LPS+IL-4 or anti-CD40+IL-4 in the presence of Zymosan, HKCA yeast or HKCA hyphae. **(A)** IgG1 class switching. Percentage of IgG1-positive cells among live B cells was measured by flow cytometry after 3 and 5 days of culture. For representative plots and gating strategy see **Supplementary Figure S1C**. **(B)** IgG1 production. The concentration of secreted IgG1 in cell culture supernatants was measured after 3 and 5 days of culture by ELISA. Data represent mean ± Standard error of the mean (SEM) of 10 mice, with triplicate measurements performed for each mouse. *p < 0.05, **p < 0.005, ***p < 0.001.

To assess how *Candida albicans* contributes to B cell activation, we isolated murine splenic B cells by anti-CD43 MACS depletion for *in vitro* stimulation. B cell purity was generally above 95%. As a baseline for testing different fungal preparations, we used either culture in medium alone, stimulation with LPS and IL-4 to mimic a T cell-independent B cell response, or stimulation with -anti-CD40 and IL-4 to mimic T cell-dependent B cell stimulation. Survival of B cells upon 3-5 days of culture in medium alone was much improved when basal stimulation was added (**Supplementary Figure S1A**). The presence of Heat killed *C. albicans* (HKCA) yeast and hyphae showed only moderate effects on class switch recombination to IgG1 (**Supplementary Figure S1C**, **Figure 1A**), but hyphae significantly increased IgG1 secretion in case of basal anti-CD40+IL-4 stimulation (**Figure 1B**). Hyphae also significantly stimulated IgM secretion in these experiments (**Supplementary Figure S1B**). Since hyphae but not yeast cells were able to stimulate B cells in this context, we reasoned that hyphae-specific components are involved. During morphogenesis to hyphae, cell wall components of the fungus become exposed. Accordingly, the fungal cell wall preparation Zymosan showed similar effects to *C. albicans* hyphae in B cell stimulation with anti-CD40+IL-4 (**Figures 1A, B**), implying that exposure of cell wall components during hyphae formation of *C. albicans* is capable of direct stimulation of B cells.

**Figure 2:**
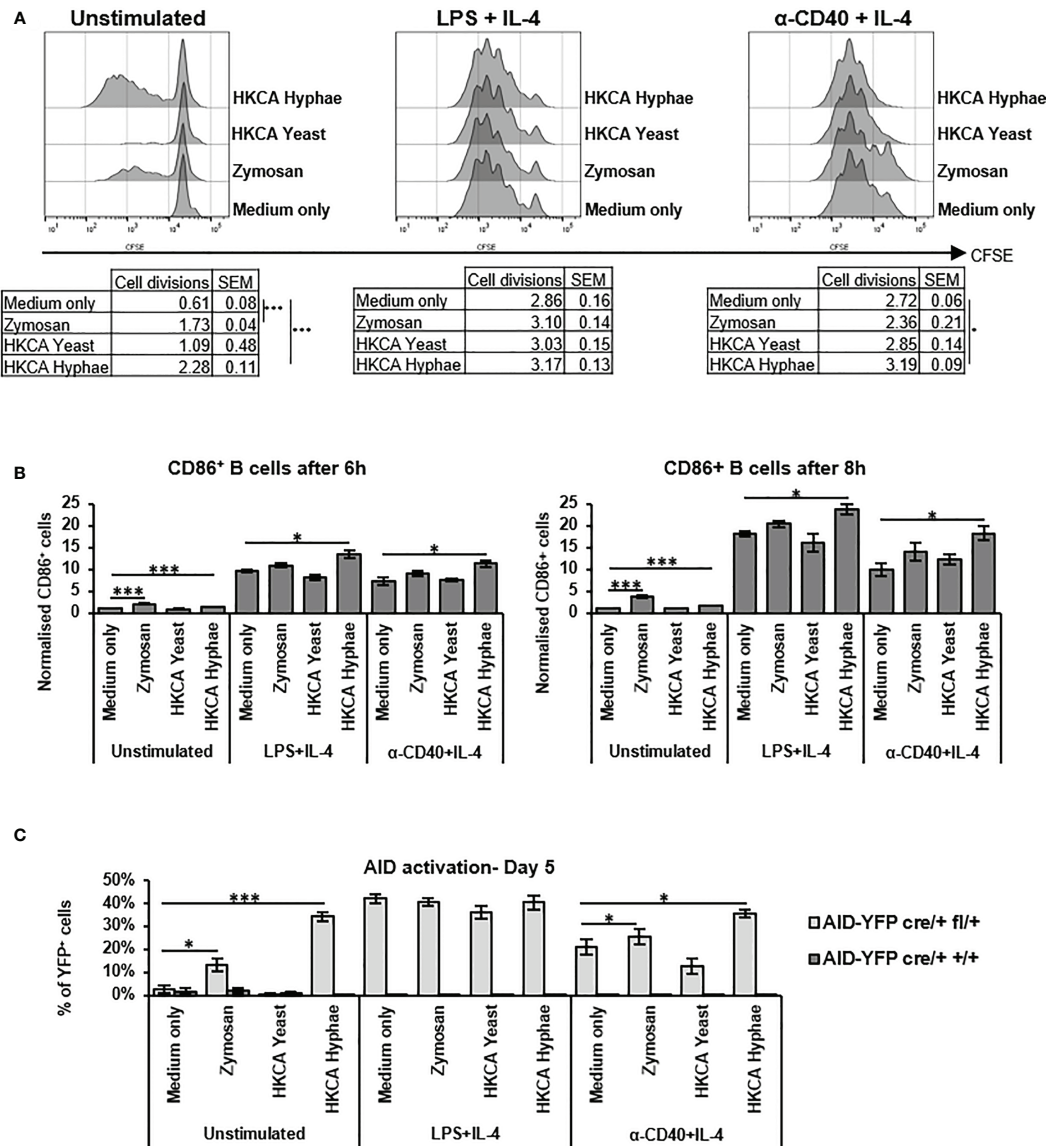
Zymosan and HKCA hyphae lead to increased B cell activation. Splenic mouse B cells were cultured either unstimulated or stimulated with LPS+IL-4 or anti-CD40+IL-4 in the presence of Zymosan, HKCA yeast or HKCA hyphae. **(A)** B cell proliferation. Cells were stained with CFSE before culture and B cell proliferation was measured for 4 days by flow cytometry based on CFSE dilution. Representative plots from day 4 are shown. Tables show number of cell divisions in 4 days. **(B)** CD86 expression. CD86 expression was measured by flow cytometry 6 and 8 hours after stimulation. For combination of data from 3 experiments, the percentage of CD86+ cells in cells cultured unstimulated with medium only was set to 1. For representative plots and gating strategy, see **Supplementary Figure S1D**. **(C)** AID activation. The percentage of YFP-positive cells within live B cells was measured by flow cytometry after 5 days of culture. For representative plots and gating strategy, see **Supplementary Figure S2B**. Data represent mean ± SEM of 3 mice, with triplicate measurements performed for each mouse. *p < 0.05, ***p < 0.001.

In order to assess whether increased IgG1 secretion is due to formation of more secreting cells by enhanced proliferation, or to increased activation of the B cells for secretion, we labelled cells with CFSE and monitored their proliferation in the presence of Zymosan, *C. albicans* yeast or hyphae. While Zymosan or hyphae were able to trigger B cell proliferation on their own, in case of basal anti-CD40+IL-4 stimulation, proliferation of the cells was only very moderately increased in the presence of *C. albicans* hyphae (**Figure 2A**). This suggests that the enhancement of IgG1 production under these conditions is not solely due to enhanced cell proliferation but might involve increased cell activation. Indeed, after stimulation with (Zymosan or) hyphae, we detected an increased number of cells with high expression of CD86 on their surface (**Figure 2B** and **Supplementary Figures S1D, E**), indicative of enhanced activation of the cells *via* NFkB which is required for CD86 expression.

To assess whether different *Candida* species elicited a differential B cell response, we tested *C. glabrata* and *C. auris* (which both only form yeast cells) for stimulation of IgG secretion by B cells. Neither of these two elicited an increased response, indicating that the B cell focus on hyphal forms leads to a differential recognition of *Candida* species by B cells. (**Supplementary Figure S2**).

To corroborate the findings of higher activation of B cells by *C. albicans* hyphae, we used a mouse model in which activated B cells turn yellow by AID-Cre mediated excision of a stop cassette in a Rosa26-stop-YFP reporter gene (**Supplementary Figure S3A**). More YFP-positive B cells were detected when cells were stimulated with Zymosan or *C. albicans* hyphae, indicative of enhanced B cell activation and hence AID expression (**Figure 2C** and **Supplementary Figure S3B**). We conclude that *C. albicans* hyphae but not yeast, as well as Zymosan, can directly activate murine B cells and stimulate IgG1 secretion.

### Dependence of B Cell Activation Upon Hyphal Stimulation on MyD88 and TLR2

**Figure 3:**
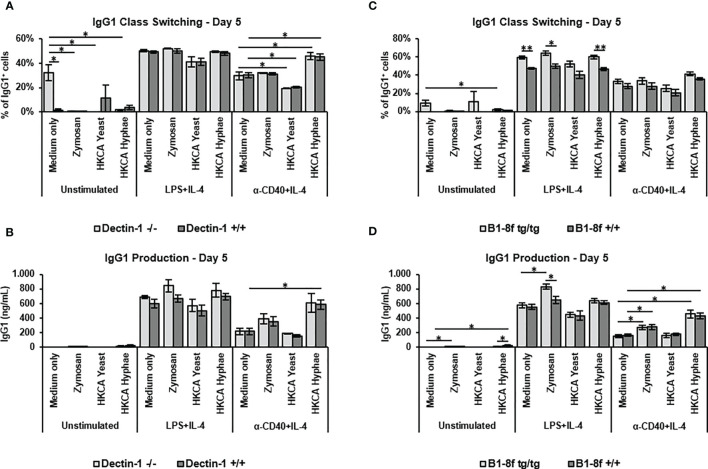
Increased antibody production upon stimulation with Zymosan or HKCA hyphae is not dependent on dectin-1 or specific B cell receptor recognition. Splenic B cells from Dectin-1^-/-^ and Dectin-1^+/+^ mice **(A, B)** and B1-8f^tg/tg^ and B1-8f^+/+^ mice **(C, D)** were cultured unstimulated or stimulated with LPS+IL-4 or anti-CD40+IL-4 in the presence of Zymosan, HKCA yeast or HKCA hyphae. After 5 days of culture, the percentage of IgG1-positive cells within live B cells was measured by flow cytometry **(A, C)**, and the concentration of secreted IgG1 was measured by ELISA **(B, D)**. Data represent mean ± SEM of 3 mice, with triplicate measurements performed for each mouse. *p < 0.05, **p < 0.005.

Previous studies on B cell stimulation by *C. albicans* have implicated either Dectin-1 stimulation by fungal cell wall components (44), or antigen specific signals that would be transmitted through the B cell receptor (34). Although low level expression of the *clec7a* gene coding for Dectin-1 could be detected in B cells by RT-PCR (**Supplementary Figure S4A**), flow cytometry analyses showed no evidence of Dectin-1 expression on murine B cells (**Supplementary Figure S4B**). Noteworthy is the fact that unspecific Dectin-1 staining was seen, also in Dectin-1 knockout cells, despite the blocking of Fc receptors or the usage of different anti-dectin-1 antibody clones (**Supplementary Figure S4B**). Importantly, though, stimulation of B cells from Dectin-1 knockout mice showed effects identical to wildtype controls of both *C. albicans* hyphae as well as Zymosan in all experimental settings (**Figures 3A, B**), indicating that Dectin-1 plays no role in the stimulation of IgG1 production by murine B cells under our experimental conditions.

To analyze the role of antigen recognition by the B cell receptor, we used B cells from B1-8f mice, which express an identical B cell receptor specific for the NP hapten (45). Stimulation of B1-8f B cells by *C. albicans* hyphae and Zymosan was similar to that of B cells from wildtype littermates (**Figures 3C, D**), implying that antigen recognition is not required for HKCA-enhanced B cell activation under our experimental conditions.

**Figure 4:**
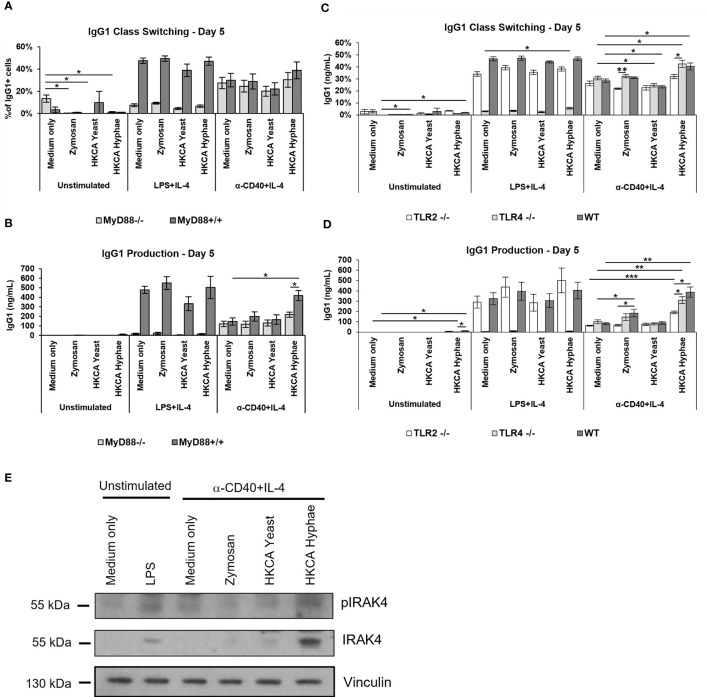
Increased antibody production upon stimulation with Zymosan or HKCA hyphae is mostly mediated *via* TLR2 recognition in an MyD88-dependent manner. Splenic B cells from MyD88^-/-^ and MyD88^+/+^ mice **(A, B)** and TLR2^-/-^, TLR4^-/-^ and wildtype (WT) control mice **(C, D)** were cultured unstimulated or stimulated with LPS+IL-4 or anti-CD40+IL-4 in the presence of Zymosan, HKCA yeast or HKCA hyphae. After 5 days of culture, the percentage of IgG1-positive cells within live B cells was measured by flow cytometry **(A, C)**, and the concentration of secreted IgG1 was measured by ELISA **(B, D)**. Data represent mean ± SEM of 4 mice per genotype, with triplicate measurements performed for each mouse. Since cells lacking MyD88 and TLR4 expression cannot recognize LPS, in cells stimulated with LPS+IL-4 the difference between MyD88-/- or TLR4^-/-^ and WT cells is statistically significant for all samples. **(E)** Western Blot analysis for the expression of phosphorylated IRAK4 (pIRAK), IRAK4 and vinculin in B cell lysates after 12 hours of stimulation. *p < 0.05, **p < 0.005, ***p < 0.001.

Antigen-independent B cell stimulation resembles a T-independent type 1 response, which depends on signaling *via* Toll-like receptors. We therefore analysed splenic B cells from mice deficient for the adaptor protein MyD88, which mediates signaling downstream of most TLRs. As expected, LPS stimulation was defective in MyD88-deficient mice (**Figures 4A, B**), as LPS signals are transmitted through TLR4/MyD88. Notably, in case of basal CD40+IL-4 stimulation, B cells from MyD88 knockout mice showed no increase in stimulation *via* Zymosan, and a significantly diminished increase in stimulation by *C. albicans* hyphae (**Figures 4A, B**). To confirm this finding by an independent method, we detected phosphorylation and accumulation of IRAK-4, a signaling intermediate directly downstream of MyD88, by Western Blotting. Both indicators of IRAK-4 signaling (46, 47) were detectable in samples stimulated with *C. albicans* hyphae (**Figure 4E**). These findings imply that Toll-like receptor signaling *via* MyD88/IRAK-4 is involved in the stimulation of murine B cells by *C. albicans* hyphae.

To identify the Toll-like receptor involved in this pathway, we tested mice deficient for TLR2 or TLR4, both of which have been implicated in the response of other immune cells to *C. albicans*. In case of basal CD40+IL-4 stimulation, B cells from TLR2-deficient mice showed significant defects in stimulation by *C. albicans* hyphae or fungal cell wall components (**Figures 4C, D**), while moderate effects seen in TLR4-deficient mice did not reach significance. We conclude therefore that upon stimulation of murine B cells with Zymosan or hyphae, TLR2 induces B cell stimulation *via* MyD88 for enhanced IgG1 production, while a contribution of other receptors, including TLR4, cannot be excluded at this point.

### Increased Stimulation of Human B Cells by *Candida albicans* Hyphae

**Figure 5:**
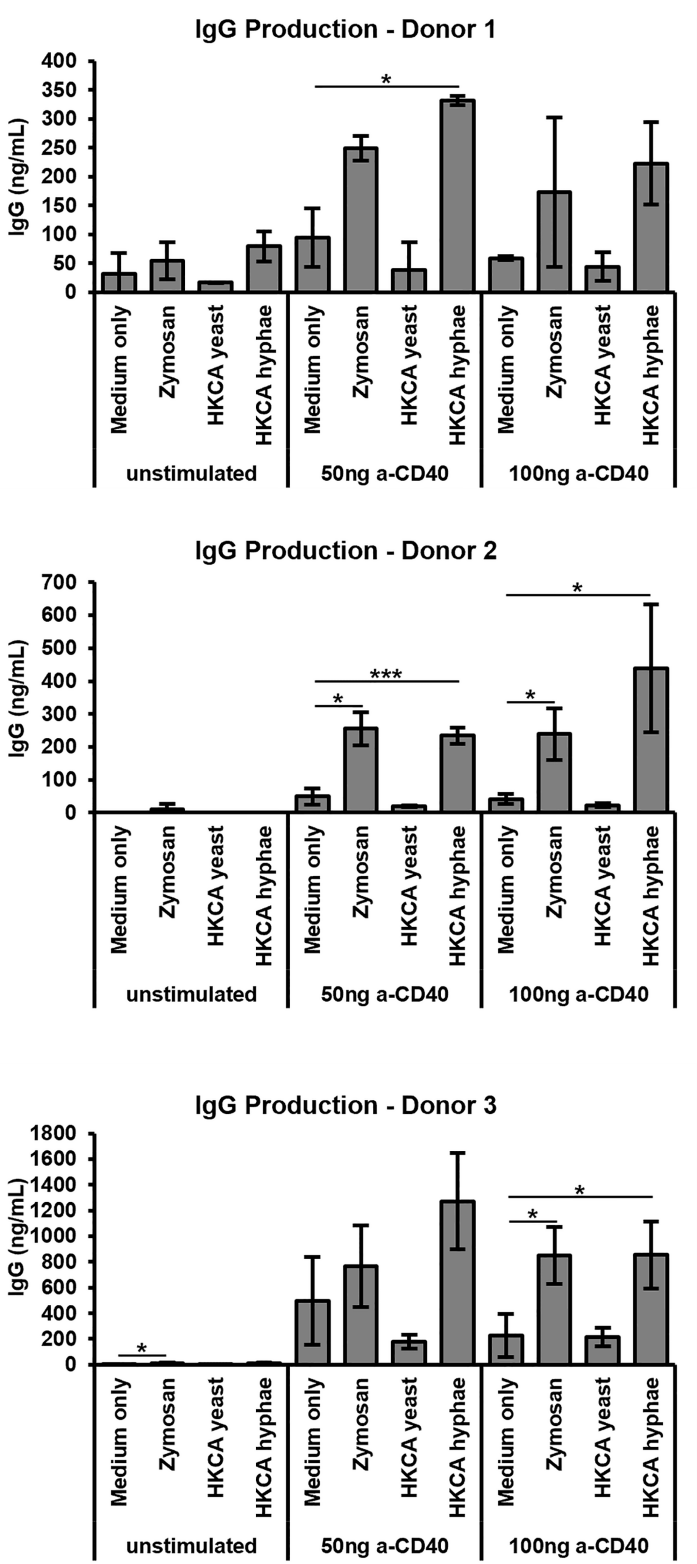
Stimulation of human B cells with Zymosan and HKCA hyphae leads to increased antibody production. B cells from human peripheral blood were cultured for 6 days unstimulated or stimulated with different anti-CD40 concentrations, anti-IgA/IgG/IgM, IL-2 and IL-10 in the presence of Zymosan, HKCA yeast or HKCA hyphae. For each donor, samples were analysed in triplicates. Data represent mean ± standard deviation (SD). *p < 0.05, ***p < 0.001.

B cells from healthy humans express little to no TLR4, but do express surface TLR2 (48). To investigate whether B cell stimulation by *C. albicans* or fungal cell wall components may also be relevant in the human, we isolated human B cells from buffy coats of healthy volunteers to a purity of >95%, stimulated them *via* the B cell receptor and CD40 in the presence of cytokines, and assessed IgG antibody production. Once again Zymosan as well as heat-killed *C. albicans* hyphae, but not yeast cells, led to stimulation of antibody secretion by the B cells (**Figure 5**), implying that our findings can be extrapolated to humans.

### Effect of Hyphal Stimulation on Cytokine Responses

**Figure 6:**
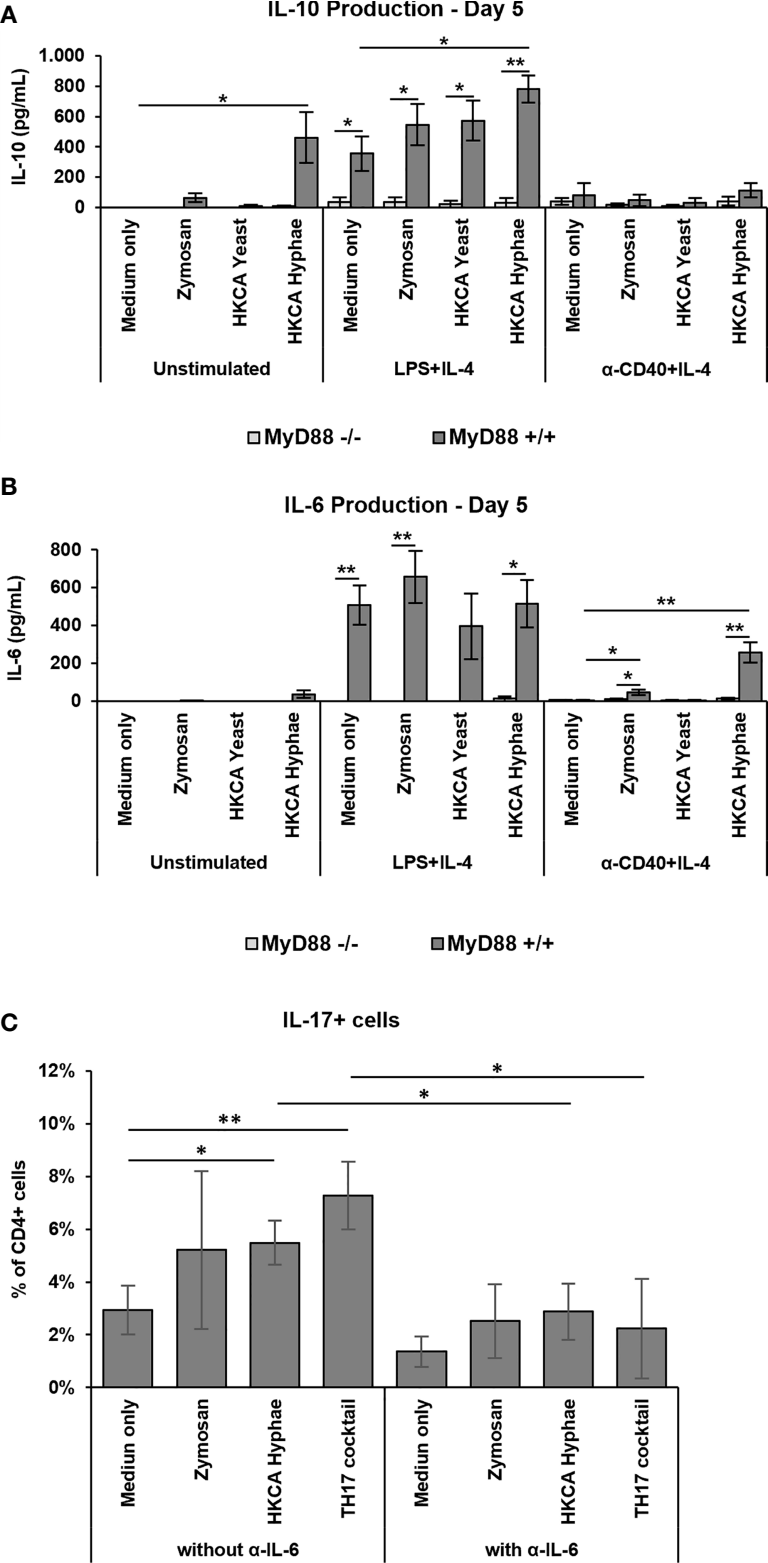
Increased MyD88-dependent IL-6 production and IL-17 expression and T_H_17 differentiation. Splenic B cells from MyD88^-/-^ and MyD88^+/+^ mice were cultured either unstimulated or stimulated with LPS+IL-4 or anti-CD40+IL-4 in the presence of Zymosan, HKCA yeast or HKCA hyphae **(A, B)**. After 5 days of culture, the concentration of secreted IL-10 **(A)** and IL-6 **(B)** was measured by ELISA. Data represent mean ± SEM of 4 mice per genotype, with triplicate measurements performed for each mouse. **(C)** Splenic B and T cells from wildtype mice were cocultured with anti-CD3ϵ and anti-IgM in the presence of Zymosan, HKCA hyphae or a T_H_17 differentiation inducing cocktail (anti-IL-4, anti-INF-γ, anti-IL-12, TGF-β1 and IL-6). The same culture conditions were also used in combination with a neutralizing IL-6 antibody. The percentage of IL-17+ cells among CD4+ T cells was measured by flow cytometry after 4 days of culture. For representative plots and gating strategy, see **Supplementary Figure S5D**. Data represent mean ± standard deviation (SD) of 3 mice with duplicate measurements performed for each condition. *p < 0.05, **p < 0.005.

To investigate the effect of MyD88-mediated B cell activation on cytokine responses, we performed a Legendplex screen for cytokine secretion into the culture medium. While IL-2, 5 and 13 were not detectable, TNF-α, IFN-γ and IL-4 showed variable secretion in the different stimulation conditions (**Supplementary Figure S5A**). IL-10 and IL-6 showed interesting secretion patterns, which were reinvestigated by ELISA. Furthermore, the IL-6 secretion of B cells was confirmed using an intracellular staining for the cytokine (**Supplementary Figure S5B**). Both cytokines were strongly induced upon LPS+IL-4 stimulation (**Figures 6A, B**), with hardly any additional effect of fungal components, while differential induction by fungal components was observed in cells without basal stimulation or CD40+IL-4 stimulation. IL-10 production was induced by HKCA hyphae without basal stimulation but hardly detectable upon CD40+IL-4 stimulation (**Figure 6A**). Notably, IL-6 secretion, which was hardly detectable without basal stimulation, was clearly induced by Zymosan and HKCA hyphae upon basal CD40+IL-4 stimulation (**Figure 6B**). Clearly, both IL-10 and IL-6 production were abolished in the absence of MyD88 (**Figures 6A, B**), implying that Toll-like receptor signaling is essential for cytokine production in this context.

### Induction of T_H_17 Differentiation Upon B Cell Stimulation With *Candida albicans* Hyphae

IL-6 production by human B cells stimulated with *C. albicans* has recently been shown to stimulate T_H_17 differentiation of T cells (34). To investigate whether the IL-6 produced in our experiments by murine B cells has the same effect, we cocultured murine B cells stimulated by Zymosan or HKCA hyphae with murine T cells and determined the efficiency of T_H_17 differentiation in the presence or absence of a neutralizing anti-IL6 antibody. Indeed, B cells stimulated with *C. albicans* hyphae led to more T_H_17 cells (**Figure 6C** and **Supplementary Figure S5D**), providing a functional link of enhanced B cell responses to *C. albicans* to protective T cell responses. This effect was abrogated when a neutralizing anti-IL-6 antibody was added to the cultures, indicating that induction of T_H_17 differentiation by hyphae-treated B cells is IL-6 dependent.

## Discussion

In the present study, we show that mouse and human B cells can be directly stimulated by fungal components, specifically *C. albicans* hyphae and the fungal cell wall preparation Zymosan, and react by enhanced antibody secretion. Stimulation led to greater activation and slightly enhanced proliferation of the cells, and did not occur *via* Dectin-1 or B cell receptor signals, contrary to previous claims (34, 44). Rather, MyD88 stimulation *via* TLR2 was mostly found responsible. Stimulation by fungal components led to increased secretion of the proinflammatory cytokine IL-6, which promoted TH_17_ differentiation.

Enhanced stimulation of the B cells by fungal components was seen in some experiments in the absence of additional stimulation, but was most clearly detectable for all readouts in the samples stimulated with CD40 and IL-4. For samples stimulated with LPS and IL-4, we did not see enhancement of stimulation by fungal components, likely due to the high basal activation triggered by LPS *via* TLR signaling. Our findings indicate that TLR/MyD88 signals triggered by fungal components cooperate with CD40-mediated costimulation of B cells. It will be highly interesting to investigate whether TLR signaling is activated in B cells by fungal components *in vivo*, and if it can enhance T cell-dependent activation of antifungal immune responses.

The stimulation with *C. albicans* hyphae led to enhanced activation of the B cells, indicated by CD86 and AID expression, and to slightly enhanced proliferation. Both higher cell activation and higher cell numbers may contribute to enhanced secretion of IgG1 and IgM by the B cells. The stimulation conditions we have used are not sufficient for plasma cell generation, but enhanced IgG1 and IgM secretion nonetheless indicates the activation of a program that pushes the cells towards a plasmablast fate. The most likely transcription factor involved is NFkB, which is required for CD86 upregulation and IL-6 secretion and is triggered downstream of TLR/MyD88 signaling.

We have shown that the stimulation effect seen in this study was independent of Dectin-1 and B cell receptor signals. Previous studies using different stimulation conditions [i.e. *C. albicans* yeasts (44) or protein extracts (34)] claimed direct B cell stimulation by Dectin-1 mediated recognition of β-glucans, or by antigen-dependent stimulation, which would require the BCR. Experimental differences are evident, as the first study was performed by LPS-only stimulation of mouse B cells, while the second study involved human B cells. However, none of these studies supported their claim by direct genetic testing of the influence of Dectin-1 and the B cell receptor, respectively, as we have done. It should be noted, too, that the expression of Dectin-1 by mouse B cells is debatable (49), and also not clearly detectable in our study, while human B cells express Dectin-1 (50). Importantly, B cell receptor-independence of the signaling implies T independent type 1 signaling in B cells, which is BCR independent and relies on Toll-like receptors.

We indeed show that the stimulation detected in our study is dependent on MyD88 signaling, and triggered mostly by TLR2. TLR2 has been implicated in *C. albicans* recognition before in myeloid cells, where it contributes to cell activation (19). The receptor is best known for the recognition of phospholipomannan cell wall components of *C. albicans*, and it signals as a heterodimer together with TLR1 and TLR6 (19). At present, we do not know the molecular basis of differential activation of B cells by yeast and hyphal forms of *C. albicans*, as both preparations contain phospholipomannan. Potentially, this component is differentially exposed in hyphae *versus* yeast, leading to differences in its recognition by B cells. Notably, even in the absence of TLR2, *C. albicans* hyphae triggered higher stimulation of B cells, implying that hyphae are recognized as well by different receptors. Which components and receptors are also involved in this recognition remain to be defined.

A very notable finding of our study is enhanced IL-6 secretion of B cells upon stimulation by fungal components. IL-6 contributes to plasma cell differentiation of B cells as well as to T_H_17 differentiation of T cells. Accordingly, we show in coculture experiments that the B cells stimulated with *C. albicans* hyphae lead to increased T_H_17 differentiation in an IL-6-dependent manner. This indicates that stimulation by fungal components, i.e. hyphae or cell wall components, *via* TLRs/MyD88, instigates B cells to produce IL-6 which will contribute to T_H_17 differentiation for fungal clearance. Interestingly, CD40 stimulation not only allowed for proinflammatory IL-6 secretion but at the same time decreases hyphae-induced anti-inflammatory IL-10 secretion, paving the way for an inflammatory T_H_17 response.

In sum, we show that B cells directly react to fungal components, in particular pathogenic hyphae, and become activated for higher antibody production and IL-6 secretion, a cytokine required for differentiation of T cells towards a T_H_17 fate for fungal clearance. It will be highly interesting to investigate the relevance of this mechanism for antifungal immune responses *in vivo*.

## Data Availability Statement

The original contributions presented in the study are included in the article/**Supplementary Material**. Further inquiries can be directed to the corresponding author.

## Ethics Statement

The studies involving human participants were reviewed and approved by Ethics committee of the University Hospital Jena. Written informed consent for participation was not required for this study in accordance with the national legislation and the institutional requirements. The animal study was reviewed and approved by Thueringer Landesamt für Verbraucherschutz.

## Author Contributions

MF-G performed most experiments and wrote the manuscript. MW performed experiments with human B cells, co-culture of murine B and T cells, and Western Blot analyses. SB analyzed CD86 and AID expression. BH and IJ provided resources and discussed data. BJ designed and supervised the entire study and wrote the manuscript. All authors read and approved the manuscript.

## Funding

BJ, IJ, and BH were supported by the Collaborative Research Centre/Transregio 124 “FungiNet” (DFG project number 210879364, projects C1, C4 and C5) of the Deutsche Forschungsgemeinschaft. MF-G was supported by a PhD fellowship of the Jena School for Microbial Communication.

## Conflict of Interest

The authors declare that the research was conducted in the absence of any commercial or financial relationships that could be construed as a potential conflict of interest.

## Publisher’s Note

All claims expressed in this article are solely those of the authors and do not necessarily represent those of their affiliated organizations, or those of the publisher, the editors and the reviewers. Any product that may be evaluated in this article, or claim that may be made by its manufacturer, is not guaranteed or endorsed by the publisher.
