# Supplementary material for: B Cell Recognition of Candida albicans Hyphae via TLR 2 Promotes IgG1 and IL-6 Secretion for TH17 Differentiation
Source: Front Immunol. 2021 Nov 8;12:698849. doi: 10.3389/fimmu.2021.698849 (PMC8606576; doi:10.3389/fimmu.2021.698849)
Supplement: Supplementary file 1 [file DataSheet_1.pdf]

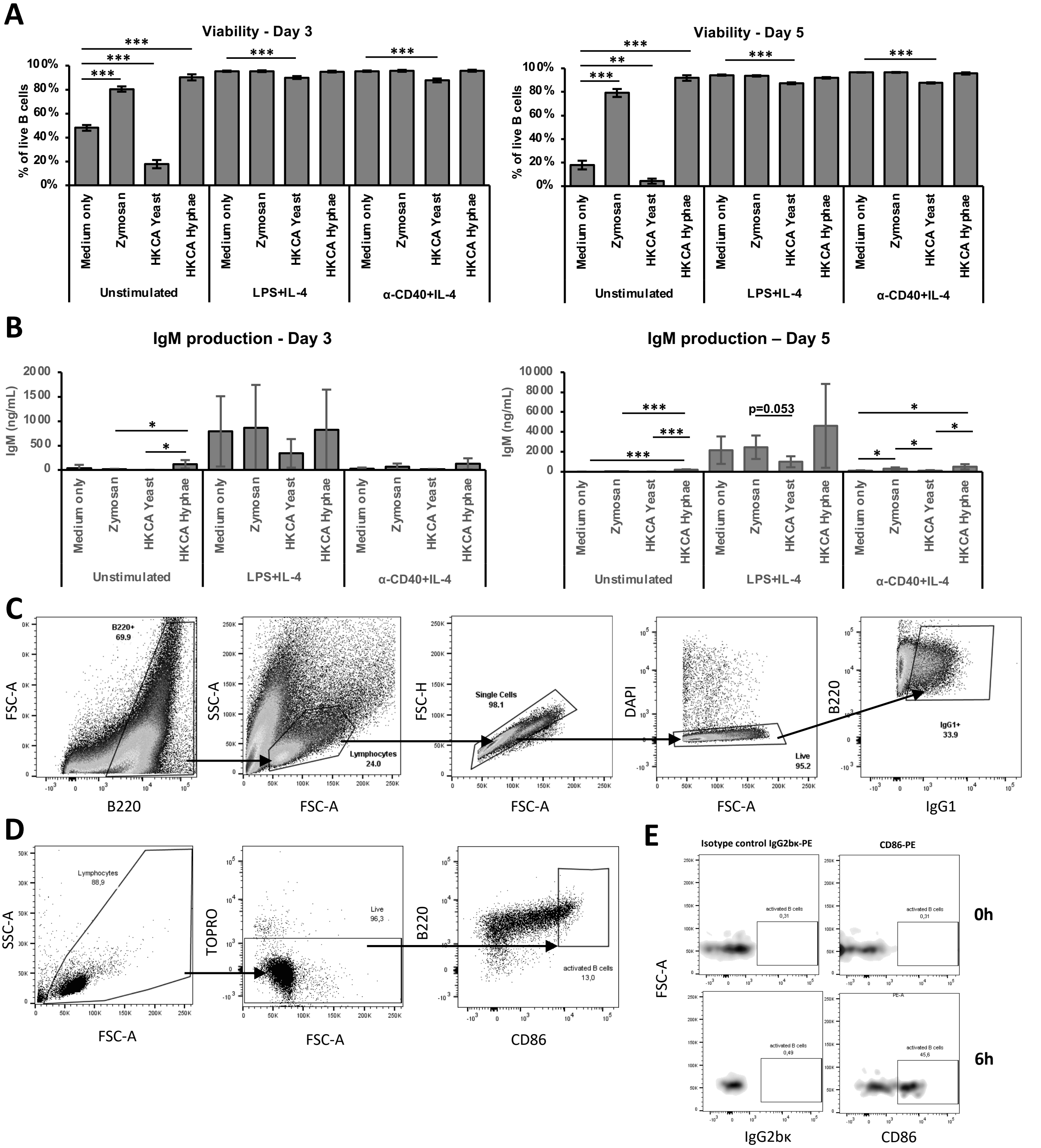

Fig S1. Cell viability, IgM production and gating strategy. Splenic mouse B cells were isolated by MACS and cultured (5x10^5 cells/mL) for 5 days, either unstimulated or stimulated with LPS+IL-4 or anti-CD40+IL-4 in the presence of Zymosan, HKCA yeast or HKCA hyphae. (A) On day 3 and 5 of culture, viability of the cells was analysed by flow cytometry. The viability was determined by percentage of living (DAPI negative) cells among B220+ single lymphocytes. (B) Splenic mouse B cells were cultured either unstimulated or stimulated with LPS+IL-4 or anti-CD40+IL-4 in the presence of Zymosan, HKCA yeast or HKCA hyphae. The concentration of secreted IgM in cell culture supernatants was measured after 3 and 5 days of culture by ELISA. Data represent mean ± Standard error of the mean (SEM) of 4 mice, with triplicate measurements performed for each mouse. *p<0.05, **p<0.005, ***p<0.001 (C) Gating strategy for viability assessment and IgG1 class switch recombination analysis. On day 3 and 5 of culture, the cells were analysed by flow cytometry to determine the percentage of IgG1-positive cells. B cells were first gated to exclude other cells or particles ($B220^+$). Debris and doublets were excluded in subsequent lymphocyte and single cell gates. Dead cells were excluded by positive DAPI staining and the percentage of $IgG1^+$ cells was obtained within this gate. Representative plots show cells stimulated with anti-CD40+IL-4 in the presence of HKCA hyphae recovered after 5 days in culture. (D) Gating strategy for CD86+ B cells. Splenic mouse B cells were isolated by MACS and cultured (5x10^5 cells/mL) for 6h or 8h, either unstimulated or stimulated with LPS+IL-4 or anti-CD40+IL-4 in the presence of Zymosan, HKCA yeast or HKCA hyphae. After 6h and 8h, the cells were analysed by flow cytometry to determine the percentage of activated B cells. After gating for lymphocytes, dead cells were excluded by positive TOPRO staining. Within this gate, the percentage of $B220^+$ $CD86^+$ cells was obtained. Representative plots show cells stimulated with LPS+IL-4 in the presence of Zymosan after 6 hours in culture. (E) Isotype control for $CD86^+$ B cells. To ensure the observed activation is due to specific antibody binding to the target, experiments were performed with an additional isotype control (IgG2b kappa-PE). Representative FACS plots show activated B cells among $B220^+$ cells directly after isolation (0h) and 6h stimulation with LPS+IL-4 in the presence of Zymosan.

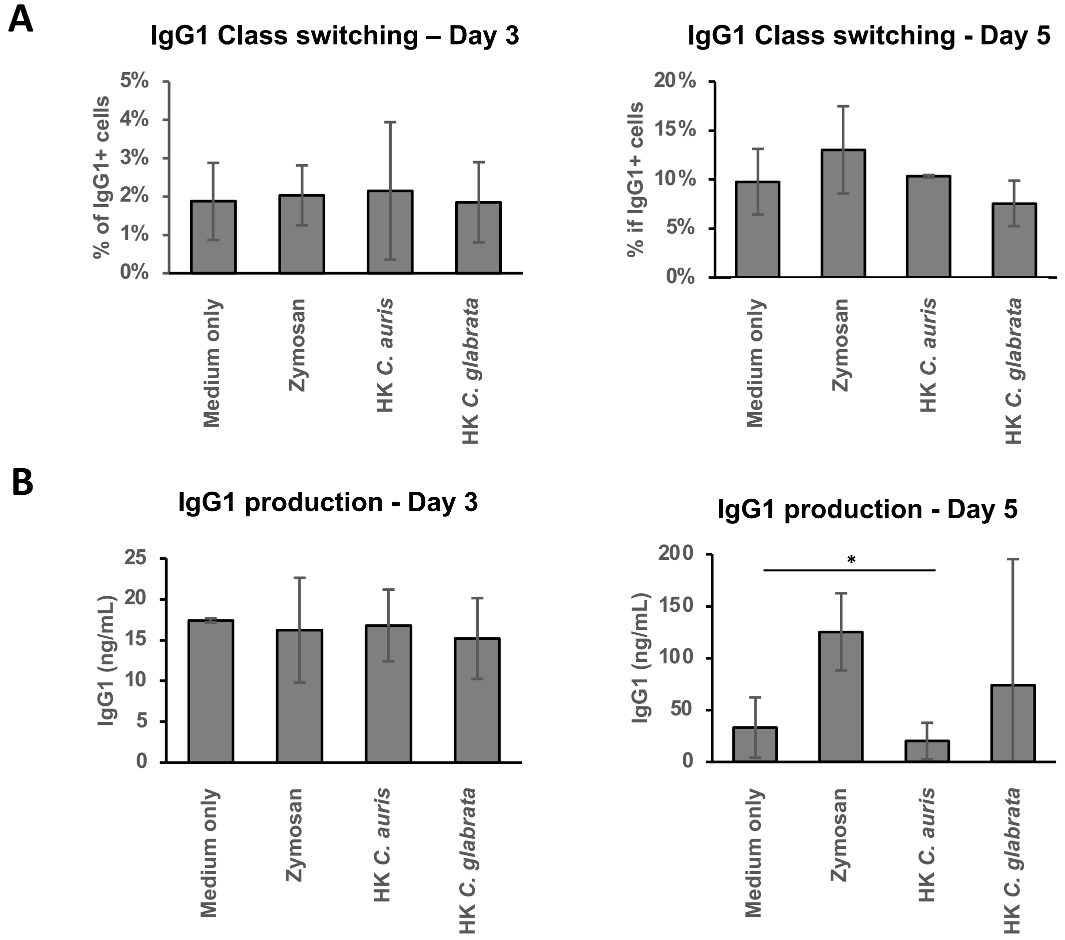

Fig. S2. B cell stimulation with heat-killed (HK) *C. auris* and *C. glabrata* yeast does not lead to increased IgG1 class switching or antibody production. Splenic mouse B cells were cultured with anti-CD40+IL-4 in the presence of Zymosan, HK *C. auris* yeast or HK *C. glabrata* yeast . (A) IgG1 class switching. Percentage of IgG1-positive cells among live B cells was measured by flow cytometry after 3 and 5 days of culture. For gating strategy see Figure S1C. (B) IgG1 production. The concentration of secreted IgG1 in cell culture supernatants was measured after 3 and 5 days of culture by ELISA. Data represent mean ± Standard error of the mean (SEM) of 3 mice, with triplicate measurements performed for each mouse. *p<0.05, **p<0.005, ***p<0.001

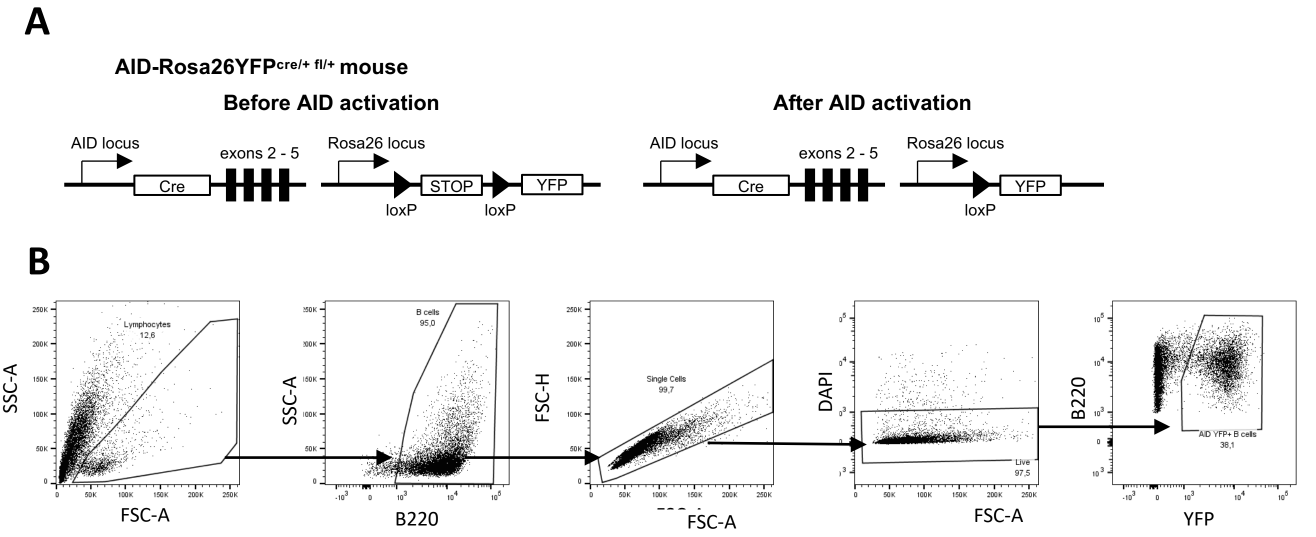

Fig. S3. Scheme and gating strategy of AID-Rosa26YFP$^{cre/+\ fl/+}$ mice. (A) Schematic representation of the modified loci in AIDCre-Rosa26YFP mice before and after AID activation. Splenic B cells from both AID-Rosa26YFP$^{cre/+\ fl/+}$ and AID-Rosa26YFP$^{cre/+\ +/+}$ mice were used. (B) Gating strategy for AID activation analysis. Splenic mouse B cells were isolated by MACS and cultured ($5x10^5$ cells/mL) for 3 or 5 days, either unstimulated or stimulated with LPS+IL-4 or anti-CD40+IL-4 in the presence of Zymosan, HKCA yeast or HKCA hyphae. On day 3 and 5 of culture, the cells were analysed by flow cytometry to determine the percentage of YFP-positive cells. B cells were gated among lymphocytes. B cells were subsequently gated for single cells and dead cells were excluded by positive DAPI staining. The percentage of B220$^+$ YFP$^+$ cells was obtained within this gate. Representative plots show cells stimulated with LPS+IL-4 in the presence of Zymosan recovered after 5 days in culture.

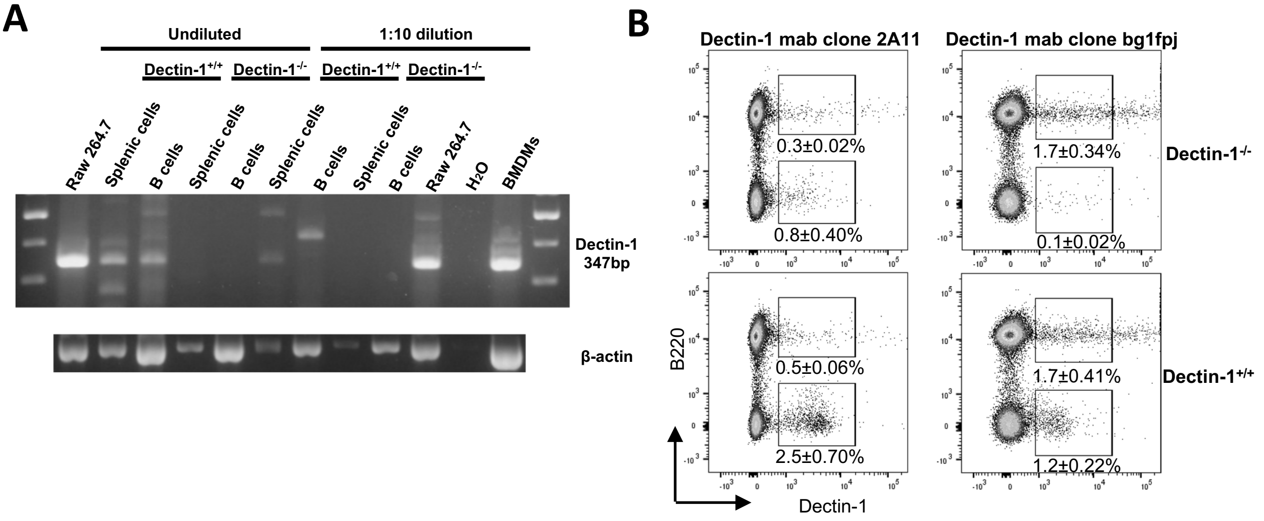

Fig S4. Dectin-1 expression in murine B cells. (A) The presence of dectin-1 mRNA was analysed by RT-PCR in splenic and B cells from Dectin-1$^{-/-}$ and Dectin-1$^{+/+}$ mice, the macrophage cell line RAW 264.7 and bone marrow-derived macrophages (BMDMs). β-actin was used as a loading control. (B) The expression of dectin-1 on the surface of mouse B cells was analysed by flow cytometry using two different antibody clones - bg1fpj (PE-Cy7) and 2A11 (FITC). The populations of interest were gated after exclusion of debris, dead cells and doublets. The Dectin-1 gating strategy was chosen based on the population of Dectin-1-positive cells (non-B cells) stained with the anti-Dectin-1 antibody clone 2A11. Representative dot plots are shown. Data represent mean ± SD of 3 mice per genotype.

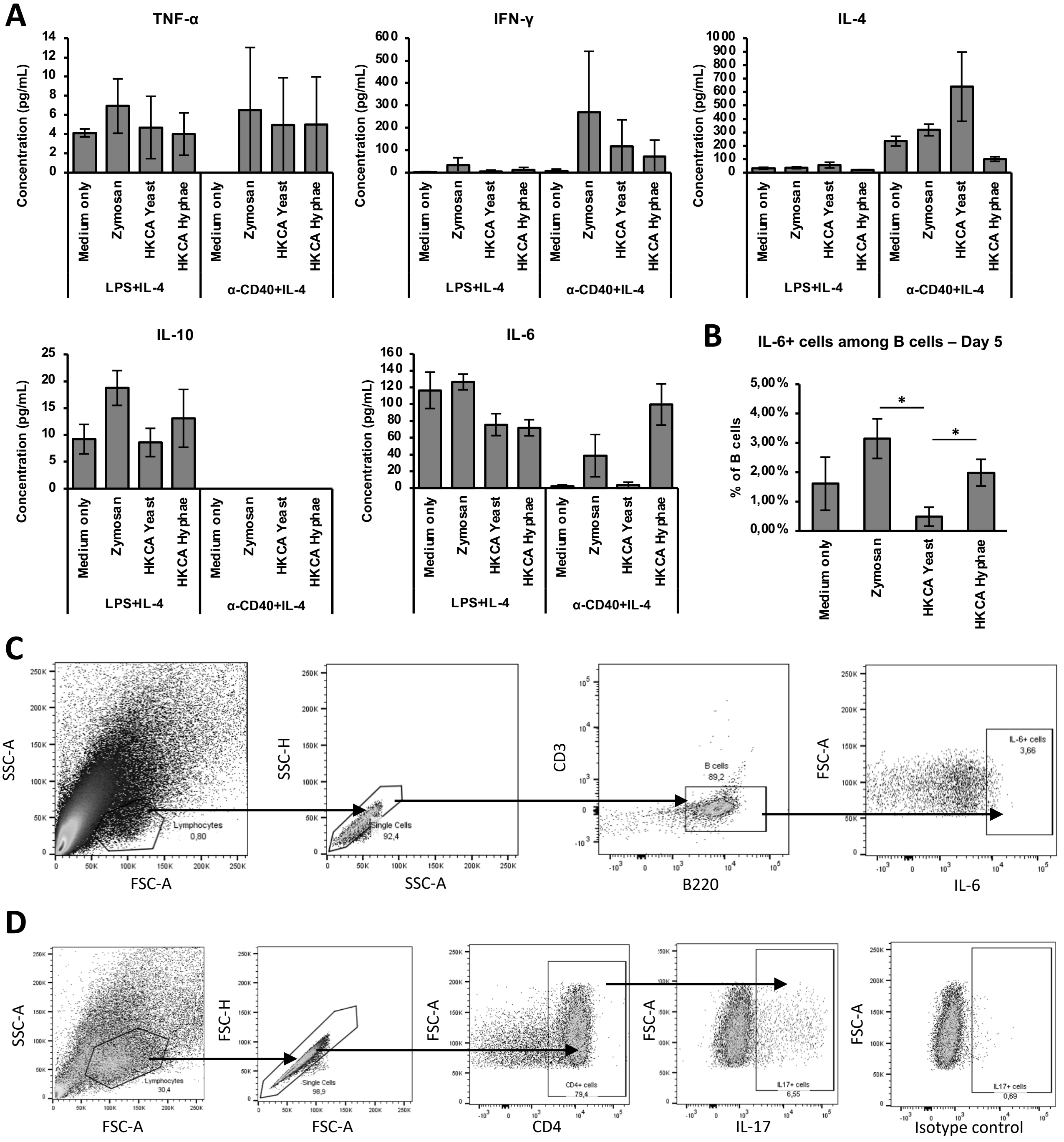

Fig S5. Screening of cytokines produced by B cells and gating strategy of T_H17 differentiation experiments. (A) Splenic B cells from wildtype mice were stimulated for 5 days with LPS+IL-4 or anti-CD40+IL-4 in the presence of Zymosan, HKCA yeast or HKCA hyphae. Supernatants recovered after 5 days of culture were screened for differences in their secreted cytokine profile using the bead-based immunoassay LEGENDplex™. Among the tested cytokines (TNF-α, IFN-γ, IL-2, IL-5, IL-4, IL-6, IL-10 and IL-13) no IL-2, 5 or 13 was detected. Data represent mean ± SEM of 3 mice, with duplicate measurements performed for each mouse. (B) Intracellular IL-6. Splenic B cells from wildtype mice were stimulated with anti-CD40+IL-4 in the presence of hyphae. Percentage of IL-6 positive cells among B cells was measured by flow cytometry after 5 days of culture in presence of Zymosan, HKCA yeast or HKCA hyphae. Data represent mean ± SEM of 3 mice, with duplicate measurements performed for each mouse. *p<0.05, **p<0.005, ***p<0.001 (C) Gating strategy for the IL-6 staining of splenic B cells. Representative FACS plots show IL-6$^+$ cells among B220$^+$ cells after 5 days of culture in the presence of Zymosan. (D) Splenic B cells and T cells from wildtype mice were stimulated for 4 days with anti-CD3e and anti-IgM in the presence of Zymosan, HKCA hyphae or a T_H17 differentiation inducing cocktail (anti-IL-4, anti-INF-γ, anti-IL-12, TGF-β1 and IL-6). The cells were analysed by flow cytometry to determine the percentage of IL-17$^+$ cells. Lymphocytes were first gated to exclude debris and particles with regard to their size and granularity. Cells were subsequently gated for single cells and CD4+ cells and the percentage of IL-17$^+$ cells was obtained within this gate. An isotype control for the IL-17 staining antibody was used to verify the analysis. Representative plots show cells after 4 days of culture in the presence of the T_H17 differentiation inducing cocktail.
